# Excess mortality attributable to AIDS among people living with HIV in high‐income countries: a systematic review and meta‐analysis

**DOI:** 10.1002/jia2.26384

**Published:** 2024-11-04

**Authors:** Adam Trickey, Julie Ambia, Robert Glaubius, Cari van Schalkwyk, Jeffrey W. Imai‐Eaton, Eline L. Korenromp, Leigh F. Johnson

**Affiliations:** ^1^ Population Health Sciences University of Bristol Bristol UK; ^2^ Center for Modeling, Planning and Policy Analysis Avenir Health Glastonbury Connecticut USA; ^3^ The South African Centre for Epidemiological Modelling and Analysis University of Stellenbosch Stellenbosch South Africa; ^4^ Center for Communicable Disease Dynamics Department of Epidemiology Harvard T.H. Chan School of Public Health Boston Massachusetts USA; ^5^ MRC Centre for Global Infectious Disease Analysis, School of Public Health Imperial College London London UK; ^6^ Data for Impact Department United Nations Programme on HIV/AIDS Geneva Switzerland; ^7^ Centre for Infectious Disease Epidemiology and Research, School of Public Health University of Cape Town Cape Town South Africa

**Keywords:** ARV, cause‐specific, death, parameters, rates, survival

## Abstract

**Introduction:**

Identifying strategies to further reduce AIDS‐related mortality requires accurate estimates of the extent to which mortality among people living with HIV (PLHIV) is due to AIDS‐related or non‐AIDS‐related causes. Existing approaches to estimating AIDS‐related mortality have quantified AIDS‐related mortality as total mortality among PLHIV in excess of age‐ and sex‐matched mortality in populations without HIV. However, recent evidence suggests that, with high antiretroviral therapy (ART) coverage, a growing proportion of excess mortality among PLHIV is non‐AIDS‐related.

**Methods:**

We searched Embase on 22/09/2023 for English language studies that contained data on AIDS‐related mortality rates among adult PLHIV and age‐matched comparator all‐cause mortality rates among people without HIV. We extracted data on the number and rates of all‐cause and AIDS‐related deaths, demographics, ART use and AIDS‐related mortality definitions. We calculated the proportion of excess mortality among PLHIV that is AIDS‐related. The proportion of excess mortality due to AIDS was pooled using random‐effects meta‐analysis.

**Results:**

Of 4485 studies identified by the initial search, eight were eligible, all from high‐income settings: five from Europe, one from Canada, one from Japan and one from South Korea. No studies reported on mortality among only untreated PLHIV. One study included only PLHIV on ART. In all studies, most PLHIV were on ART by the end of follow‐up. Overall, 1,331,742 person‐years and 17,471 deaths were included from PLHIV, a mortality rate of 13.1 per 1000 person‐years. Of these deaths, 7721 (44%) were AIDS‐related, an overall AIDS‐related mortality rate of 5.8 per 1000 person‐years. The mean overall mortality rate among the general population was 2.8 (95% CI: 1.8–4.0) per 1000 person‐years. The meta‐analysed percentage of excess mortality that was AIDS‐related was 53% (95% CI: 45–61%); 52% (43–60%) in Western and Central Europe and North America, and 71% (69–74%) in the Asia‐Pacific region.

**Discussion:**

Although we searched all regions, we only found eligible studies from high‐income countries, mostly European, so, the generalizability of these results to other regions and epidemic settings is unknown.

**Conclusions:**

Around half of the excess mortality among PLHIV in high‐income regions was non‐AIDS‐related. An emphasis on preventing and treating comorbidities linked to non‐AIDS mortality among PLHIV is required.

## 1 INTRODUCTION

Before the availability of combination antiretroviral therapy (ART) in 1996, mortality rates among people living with HIV (PLHIV) were very high and deaths were mostly AIDS‐related [1, 2]. Since then, most PLHIV have started on ART [3], with 77% of the 39.9 million PLHIV globally being on ART in 2023, substantially reducing AIDS incidence and AIDS‐related mortality [4, 5]. More effective and well‐tolerated ART regimens have become available since 1996, further reducing AIDS cases and deaths [6, 7], with UNAIDS estimating that 630,000 PLHIV died from AIDS‐related illnesses in 2023, compared with 2.1 million in 2004 [3]. Commensurately, life expectancy for PLHIV has increased, with studies in some settings estimating it to only be below that of the general population by a few years [8, 9], and the ageing population with HIV are increasingly dying from other age‐related causes, such as cardiovascular disease (CVD) and cancer [7]. Higher prevalence of substance use and comorbidities such as hepatitis C virus in PLHIV compared to the general population also contribute to disproportionately high mortality rates [10]. Evidence also suggests that PLHIV develop various non‐AIDS‐related comorbidities at faster rates than those without HIV, particularly PLHIV with unsuppressed viral loads, potentially related to consistently heightened inflammation [11, 12]. In the era of universal ART access, further addressing excess mortality requires current data on the changing causes of mortality among PLHIV.

In comparative international cause‐of‐death assessments [13, 14], the World Health Organization recommends any death be classified as AIDS‐related in which AIDS was identified as the underlying cause, using the International Classification of Diseases, Tenth Revision (ICD‐10) [13]. In many settings affected by HIV, death registration and cause of death determination are not complete [15]. Therefore, national and global HIV epidemic estimates published by UNAIDS [16] are produced using mathematical models [17] and have estimated AIDS‐related mortality as excess mortality among PLHIV measured in cohort studies [18, 19].

Excess mortality among PLHIV can be defined as the rate of mortality above that experienced by an equivalent population without HIV, usually matched by sex and age to account for much higher mortality rates at older ages. Quantifying AIDS‐related mortality by excess mortality among PLHIV is a reasonable approximation in earlier periods in the epidemic when the predominant cause of death among PLHIV was AIDS and in settings where advanced HIV disease and mortality from AIDS remains relatively high. However, recent data have indicated that, with changing distributions of causes of death since the scale‐up of ART in 1996, a sizeable percentage of this excess mortality among PLHIV may no longer be AIDS‐related [20]. For example, it was estimated that 68% of excess mortality from 2016 to 2020 among men living with HIV in Europe was due to non‐AIDS causes [20]. While no meta‐analyses have examined how much excess mortality among PLHIV is not AIDS‐related, a meta‐analysis from 2017 found a pooled percentage of overall mortality due to non‐AIDS causes was 53.0% in high‐income settings compared to 18.5% in sub‐Saharan Africa [21].

In this systematic review and meta‐analysis, we aimed to quantify the percentage of excess mortality due to AIDS, separately for PLHIV on and off ART and by UNAIDS regions.

## 2 METHODS

This review has been reported according to Preferred Reporting Items for Systematic Reviews and Meta‐Analyses (PRISMA) guidelines [22] (Table S1).

### 2.1 Search strategy

The search was performed on 22/09/2023 in Embase (via OVID) using broad search terms. These were: ((‘AIDS’ or ‘HIV’) and (‘mortality’ or ‘death’) and (‘cause’ or ‘cause‐specific’) and (‘ART’ or ‘HAART’ or ‘ARV’ or ‘antiretroviral’)).

Studies were eligible to be included if the following criteria were met: (1) they contained data on adult PLHIV (with age cut‐offs defined by the studies); (2) the study contained data on HIV/AIDS (or non‐HIV/AIDS‐related) cause‐specific mortality rates among PLHIV; and (3) the study contained age‐matched/comparator all‐cause mortality rates among people without HIV.

Studies were excluded if they were systematic reviews, meta‐analyses or modelling studies. Studies were also excluded if they contained fewer than <10 deaths overall, because cause‐specific mortality proportions calculated from such small samples would likely be unreliable. Studies in non‐English language journals were also excluded. Studies were excluded that focused on a subgroup of people, such as children, pregnant women, intravenous drug abusers and smokers (we defined this as studies that explicitly stated in the title, abstract or aims that they were focusing on one particular subgroup of PLHIV). Additionally, studies were excluded that had only one type of cause‐specific mortality, unless it was a broad category such as AIDS‐related or non‐AIDS‐related (e.g. exclude a study that only looks at cardiovascular mortality), because otherwise AIDS‐related excess mortality could not be calculated.

After the initial inclusion/exclusion criteria were applied, the studies were re‐reviewed to avoid duplication by publication type or by cohort. Where a study was available as both a conference presentation, pre‐print or a published manuscript, then the manuscript was selected. Pre‐prints were selected above conference presentations. If two conference presentations were available (and no corresponding full‐text manuscript), then the most recent was selected. Where two different studies were found from the same cohort, then the most recent study was selected. If a study from an individual cohort that participates in a cohort collaboration, as well as a study from that cohort collaboration (with overlapping years of study) were both available, then the individual cohort study was excluded to avoid duplication of data. The references of eligible studies were also searched, to identify other eligible studies not identified by the initial search. One reviewer, AT, performed all screening and applied the inclusion/exclusion criteria.

### 2.2 Data extraction

For each study, the first author, country or countries of study, years of study and the data source or cohort were extracted, where applicable. Where possible, data were stratified across different calendar year periods and by ART status. Extraction was performed by AT and checked independently by JA.

Within each study follow‐up period, the following items were extracted for PLHIV: number of persons, number of deaths, number of deaths due to each reported type of cause‐specific mortality, person‐years, all‐cause mortality rate, cause‐specific mortality rates, excess mortality rate, definition of HIV/AIDS‐related mortality, median baseline CD4 cell count, median baseline age, percentage female and percentage on ART. For people without HIV, the following items were extracted: number of persons, number of deaths, person‐years, all‐cause mortality rate, median baseline age and percentage female.

If a study reported the all‐cause mortality rate among PLHIV and a standardized mortality ratio (SMR) compared with a general population/people without HIV, then an all‐cause mortality rate for the general population was estimated by dividing the rate among PLHIV by the SMR (e.g. 118 deaths per 10,000 person‐years among PLHIV divided by an SMR of 5.7 = 20.7 deaths per 10,000 person‐years). If studies reported the total number of deaths to PLHIV and the subset of deaths due to non‐AIDS causes, then the difference was used as AIDS‐related deaths.

Each study was assessed for quality using a 9‐item tool developed for this review through amending another quality assessment tool for quantitative studies [23], shown in the Supplementary Materials. The quality assessment was carried out independently by AT and JA. The overall quality assessment scores were calculated for each study by summing the individual components. Where there was a difference in the overall score for a study of one point between the two markers, the average score was taken (rounding up). No differences of greater than one point occurred between the two markers.

### 2.3 Evidence synthesis of excess mortality

We reported the overall excess mortality rates among PLHIV compared to the general population, the AIDS‐related mortality rate among PLHIV and the proportion of excess mortality due to AIDS‐related mortality. Excess mortality among PLHIV was defined as the rate of mortality above that experienced by an equivalent population without HIV, matched by sex and age. In cases where the number of “excess deaths” was not available, it was calculated by dividing the number of deaths due to AIDS by the proportion of the excess mortality rate that was due to AIDS (e.g. 1268/0.29 = 4367). These data were meta‐analysed using the metaprop command in Stata to calculate the pooled proportion of excess mortality due to AIDS including random effects, which applies the Freeman‐Tukey double arcsine transformation. This was repeated to calculate the pooled proportion of all‐cause mortality due to AIDS. Pooled proportions were presented in forest plots. Heterogeneity was assessed using the *I* ^2^ statistic [24]. A sensitivity analysis was performed excluding studies with a quality assessment score of 6 or less (high risk of bias). We also performed a “leave‐one‐out analysis” where the pooled proportions were calculated leaving out one study at a time to identify how much the results would vary.

### 2.4 Meta‐regression

A random‐effects meta‐regression was carried out through the metareg command in Stata weighted by the number of excess deaths. The outcome was the proportion of excess mortality due to AIDS and the independent variables were study mid‐year, national ART coverage at the study mid‐year point using UNAIDS’ estimates and whether the Causes of Death in HIV (CoDe) protocol [25] was used to determine mortality due to HIV/AIDS. Univariable and multivariable models were constructed. Variables were included in the multivariable models that had *p*‐values <0.1 in the univariable models. For the study by Karnite and Brigis [26], we took 2009 as the study mid‐year as this was the year that was used to calculate the SMRs with the general population, although the study start year was 1987 when the first case of HIV in Latvia was recorded. For the multi‐country study by Trickey et al. [27], we used the national ART coverage of the country in which the most PLHIV in the study resided—France.

All analyses were performed in Stata version 18 [28].

## 3 RESULTS

### 3.1 Study characteristics

Of the 4485 studies identified by the initial search, 17 were eligible for inclusion. Searching references of the eligible studies identified two additional studies from British Columbia, Canada [29] and Denmark [30] (*N* = 19). Eleven studies were then removed that were duplicate reporting of the same data: four from the Spanish CoRIS cohort [31, 32, 33, 34], two from the Danish HIV cohort [30, 35] and one from the PISCIS cohort [36] that were part of the ART‐CC reported in Trickey et al. [27], one poster that duplicated information from an included manuscript by Croxford et al. [37] and three that were previous studies reporting on the same cohort/population as more recent eligible studies [38, 39, 40]. After these removals, eight studies remained (Table 1 and Figure S1). All studies were from high‐income countries where sex between men is the primary mode of HIV acquisition; 6/8 from the Western and Central Europe and North America (WCENA) region [26, 27, 29, 41, 42, 43] (5/6 from Europe and one from Canada), one from Japan [44] and one from South Korea [45]. All were cohort studies. All but one study [26] scored 7 out of 9 or higher on the quality assessment score. The individual quality assessment scores of each reviewer are reported in Table S2.

**Table 1:** Characteristics of included studies

First author	Location	Years	Median baseline age	% female	% on ART^a^	Comparison against general population	AIDS‐related mortality definition	Quality assessment score	Study design
Croxford [41]	England and Wales	1997–2012	34 (IQR: 28–41)	36%	66%	Age‐ and sex‐matched general population mortality rates	Death reports from HIV clinicians through routine surveillance and death auditing	9	Cohort
De Coninck [42]	Sweden	1996–2011	Mean: 37.6	36%	>56%	1:2 matching with HIV‐negative people from population register by age, sex and birth region	ICD9/ICD10. Only rates for non‐AIDS cancer, cardiovascular disease and violent deaths given; the remainder were assumed to be AIDS‐related	8	Cohort
Eyawo [29]	British Columbia, Canada	1996–2012	38 (IQR: 32–46)	20%	74%	Direct comparison with a randomly sampled (age/sex‐matched) HIV‐negative control group in region who had personal health numbers	ICD9/ICD10 categories	8	Cohort
Fontela [43]	Navarra, Spain	1999–2018	1999–2003: 37 (IQR: 34–41) 2014–2018: 47 (IQR: 40–52)	33%	1999–2003: 72% 2014–2018: 88%	Age‐ and sex‐matched general population mortality rates	HIV‐related ICD10s: B20‐B24 and R75	8	Cohort
Karnite [26]	Latvia	1987–2010	NA	NA	NA	Age‐matched general population mortality rates	HIV‐related ICD10s: B20‐B24	6	Cohort
Nishijima [44]	Tokyo, Japan	2005–2016	36 (IQR: 32–46)	8%	32% at enrolment	Age‐ and sex‐matched general population mortality rates	Adapted Cause of Death (CoDe) project protocol	8	Cohort
Park [45]	South Korea	2004–2019	Mean: 40.3	11%	91%	Age‐ and sex‐matched general population mortality rates	ICD10 categories	8	Cohort
Trickey [27]	Europe (Multi‐country)	2000–2015	37 (IQR: 31–45)	27%	100%	Age‐, sex‐ and country‐matched general population mortality rates	Adapted Cause of Death (CoDe) project protocol	7	Cohort

Abbreviations: ICD, International Classification of Disease; IQR, interquartile range; NA, not available.

^a^ People off ART at start of follow‐up could have subsequently started ART.

The percentage of PLHIV who were female was <40% in all studies (all but one reported the percentage). The percentage of PLHIV on ART was not always available or clearly reported. Only one study reported exclusively on PLHIV on ART; no studies reported excess mortality rates and cause of death for PLHIV off ART. The definition of HIV/AIDS‐related mortality and how this was captured varied between studies. Two studies used the CoDe protocol [27, 44], one used a panel of physicians [41] and the other five reported physicians assessed the underlying cause of death ICD‐10 from the death certificate information. None of the included studies reported on cause‐specific mortality in a manner that would enable the proportion of mortality due to AIDS to be adjusted to exclude deaths where data on the cause were entirely missing (distinct from unknown). De Coninck et al. [42] only reported cause‐specific deaths for non‐AIDS cancer, CVD and violent deaths, so the remainder were assumed to be HIV/AIDS‐related, although this is likely an overestimate. The median study mid‐year was 2008 (interquartile range [IQR]: 2005–2011) and the median national ART coverage among PLHIV at the time of the study mid‐year was 63% (IQR: 42–73%).

### 3.2 Overall mortality

**Figure 1:**
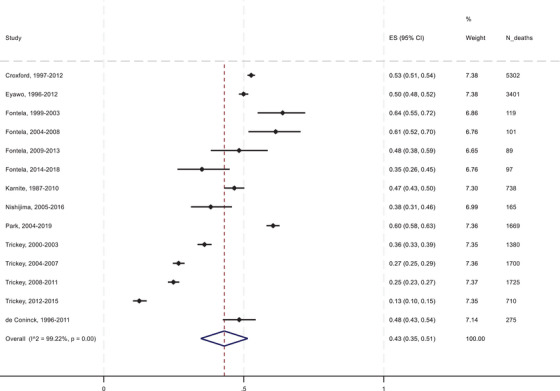
Forest plot of the pooled proportion of all mortality among PLHIV due to AIDS. ES (95% CI), pooled estimate with 95% confidence interval.

For two studies, the required data were available for multiple follow‐up periods (Table 2). The total number of person‐years among PLHIV was 1,331,742, in which time there were 17,471 deaths, for an overall mortality rate of 0.0131 per year. Of the 17,471 deaths, 7721 were classified as HIV/AIDS‐related, an overall AIDS‐related mortality rate of 0.0058 per person‐year, giving 44% of all‐cause mortality that was due to HIV/AIDS. The corresponding estimate from the random effects meta‐analysis was 43% (95% confidence interval: 35–51%) (Figure 1). Heterogeneity across the studies was very high (*I* ^2^: 99%), although the leave‐one‐out analysis (Table S3) did not show much variation in point estimates from the overall pooled analysis. When stratifying by region, the pooled percentage of overall mortality due to AIDS in the six WCENA region studies was 42% (33–51%), while in the two studies from the Asia‐Pacific region, it was 58% (56–61%).

**Table 2:** Extracted data on mortality rates

**First author**	**Years**	**Mid‐year**	**National ART coverage among PLHIV** ^**b**^	***N***	**Deaths**	**AIDS deaths**	**Person years**	**All‐cause mortality rate among PLHIV**	**AIDS mortality rate among PLHIV**	**All‐cause mortality rate among gen‐pop**	**Excess all‐cause mortality rate among PLHIV versus gen‐pop**	**AIDS mortality as % of excess mortality**	**AIDS mortality as % of all‐cause mortality**
Croxford [41]	1997–2012	2005	48%	88,994	5302	2791	448,839	0.0118	0.0062	0.0021	0.0097	64%	53%
De Coninck [42]	1996–2011	2004	42%	4066	275	133	24,336	0.0113	0.0055	0.0022	0.0091	60%	48%
Eyawo [29]	1996–2012	2005	31%	13,729	3401	1698	108,990	0.0312	0.0156	0.0086	0.0226	69%	50%
Fontela [43]	1999–2003	2001	31%	839	119	76	3552	0.0335	0.0214	0.0016	0.0318	67%	64%
	2004–2008	2006	53%	881	101	62	3826	0.0264	0.0162	0.0002	0.0246	66%	61%
	2009–2013	2011	72%	964	89	43	4159	0.0214	0.0103	0.0024	0.0190	54%	48%
	2014–2018	2016	83%	1059	97	34	4686	0.0207	0.0073	0.0028	0.0179	41%	35%
Karnite [26]	1987–2010	2009^a^	10%	4888	738	344	31,273	0.0236	0.0100	0.0021	0.0215	46%	42%
Nishijima [44]	2005–2016	2011	76%	2797	165	63	18,858	0.0088	0.0033	0.0015	0.0073	46%	38%
Park [45]	2004–2019	2012	54%	13,919	1669	1009	97,439	0.0171	0.0104	0.0030	0.0141	73%	60%
Trickey [27]	2000–2003	2002	73%	35,697	1380	496	94,735	0.0151	0.0049	0.0036	0.0115	43%	32%
	2004–2007	2006	73%	54,213	1700	454	153,993	0.0118	0.0028	0.0036	0.0082	34%	24%
	2008–2011	2010	74%	72,800	1725	429	207,739	0.0082	0.0020	0.0037	0.0045	44%	24%
	2012–2015	2014	75%	64,959	710	90	129,317	0.0049	0.0007	0.0028	0.0021	33%	14%

Abbreviation: Gen‐pop, general population.

^a^ We took 2009 as the study mid‐year as this was the year that was used to calculate the standardized mortality ratios with the general population, while the study start year was 1987 when the first case of HIV in Latvia was recorded, so the data would have been heavily right‐skewed.

^b^ Taken from UNAIDS data for corresponding mid‐year.

### 3.3 Excess mortality

**Figure 2:**
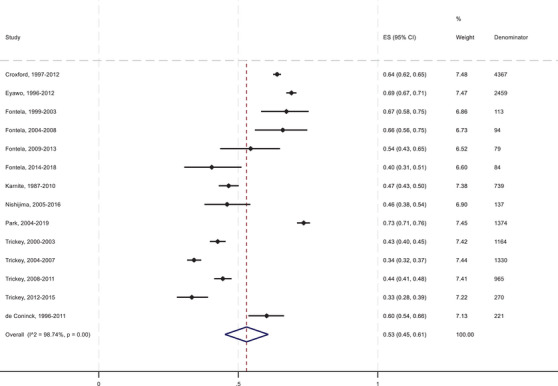
Forest plot of the pooled proportion of excess mortality among PLHIV due to AIDS. ES (95% CI), pooled estimate with 95% confidence interval.

The unweighted mean of the percentage of excess mortality attributed to HIV/AIDS‐related causes was 46%, and the meta‐analysed percentage was 53% (95% CI: 45–61%) (Figure 2). The mean mortality rate among the general population in the included studies was 0.0028 (95% CI: 0.0018–0.0040) per year. Excess mortality rates comparing the PLHIV with the general population ranged from 0.0021 in Trickey et al.’s 2012–2015 follow‐up period to 0.0318 in Fontela et al.’s 1999–2003 follow‐up period [27, 43]. The percentage of the excess mortality attributed to HIV/AIDS‐related causes ranged from 33% in Trickey et al. [27] to 73% in Park et al. [45]. In both studies that reported multiple calendar year periods, the excess mortality rate and the percentage of excess mortality attributed to AIDS decreased over time. The pooled percentage of excess mortality due to AIDS in the six WCENA region studies was 52% (43–60%), and in the two studies from the Asia‐Pacific region, it was 71% (69–74%). In a sensitivity analysis excluding the one study (Karnite and Brigis [26]) with a quality assessment score of 6 or less, the meta‐analysed percentage of excess mortality due to AIDS was 53% (95% CI: 45–62%).

### 3.4 Meta‐regression

In the multivariable meta‐regression of the proportion of excess mortality due to AIDS (Table 3), the intercept was 0.8072 (95% CI: −0.1522, 1.6297). For each additional percentage of PLHIV on ART, excess mortality due to AIDS decreased by −0.0084 (95% CI: −0.0244, 0.0075). Excess mortality due to AIDS was −0.4603 (95% CI: −1.1431, 0.2225) lower in studies using the CoDe protocol. An example of a calculation for a setting with 90% ART coverage, using the CoDe protocol would be: 1/(1+exp(−0.8072 + 0.0084×90 + 0.4603)) = 0.40. An equivalent calculation without using the CoDe protocol would be: 1/(1+exp(−0.8072 + 0.0084×90)) = 0.51. It should be noted that the confidence intervals were wide for these coefficients reflecting large uncertainty.

**Table 3:** Meta‐regression of the pooled proportion of excess mortality among PLHIV due to AIDS by ART coverage, use of the CoDe protocol and study mid‐year

	Coefficient (95% confidence interval), *p*‐value	
	Univariable	Multivariable
**ART coverage**	−0.0144 (−0.0282, −0.0007), *p* = 0.041	−0.0084 (−0.0244, 0.0075), *p* = 0.270
**Use of CoDe**	−0.6610 (−1.2252, −0.0968), *p* = 0.025	−0.4603 (−1.1431, 0.2225), *p* = 0.166
**Study mid‐year**	−0.0458 (−0.1219, 0.0304), *p* = 0.215	Not included
**Constant**	Differs by the independent variable included	0.8072 (−0.0152, 1.6297), *p* = 0.054

Abbreviation: CoDe protocol, Coding Causes of Death in HIV (CoDe).

## 4 DISCUSSION

The pooled percentage of all‐cause mortality due to AIDS from these studies, all from high‐income countries, was 43% (95% CI: 35–51%), while the pooled percentage of excess mortality among PLHIV due to AIDS was 53% (45–61%). This pooled percentage of excess mortality was calculated after subtracting the expected age‐matched mortality rates from a comparator general population. Our intention was to report and calculate these proportions separately for PLHIV on and off ART. However, no data were found for PLHIV off ART. By the end of follow‐up in all these studies, the vast majority of PLHIV will have been on ART. The pooled percentage of excess mortality due to AIDS was lower in the six studies from the WCENA region (52% [43–60%]) than in the two studies from the Asia‐Pacific region (71% [69–74%]), perhaps reflecting different levels of ART coverage or methods in assigning causes of death. That almost half of the excess mortality among PLHIV in high‐income settings is due to non‐AIDS causes is not unexpected in the era of universal availability of ART; it illustrates ART's effectiveness in reducing AIDS‐related mortality [5]. The substantial mortality unrelated to AIDS among PLHIV above the background mortality in the general population may reflect elevated risks of non‐AIDS‐related comorbidities, such as some cancers, particularly among PLHIV with worse virological status [11]. This is often seen among those who are unaware they have HIV until their immune system has been compromised and then start ART, highlighting the importance of effective HIV testing strategies. Additionally, the use of tobacco, alcohol and drugs such as opioids is more common among PLHIV than the general population, which likely contributes to some non‐AIDS‐related excess mortality [46]. Lastly, PLHIV are often from marginalized populations with lower socio‐economic status, which is a strong predictor of early mortality in populations without HIV [47]. The meta‐regression in our review indicated that the studies that used the CoDe protocol to determine causes of death had lower percentages of excess mortality due to AIDS than the studies that did not use the CoDe protocol (which mostly used ICD codes). This is potentially due to the CoDe protocol reducing the misclassification of deaths attributed to AIDS through accounting for additional information (e.g. recent CD4 cell counts) [48].

### 4.1 Comparison with other literature

This is, to our knowledge, the first review of the percentage of excess mortality among PLHIV due to AIDS. A previous review of studies up to 2015 of PLHIV on ART found that in some regions the majority of all mortality was due to non‐AIDS causes [21]. In our review, containing only studies from high‐income countries, we found that 57% (95% CI: 49–65%) of all mortality was due to non‐AIDS causes. The previous review found that in high‐income countries the pooled percentage of all mortality due to non‐AIDS causes among PLHIV on ART was similar, 53% (95% CI: 44–62%), while this was 34% (20–49%) in what they termed “developing countries,” and 19% (14–24%) in sub‐Saharan Africa [21].

### 4.2 Strengths and limitations

Strengths of our review include limiting to studies of PLHIV with age‐matched mortality information for the general population, enabling accurate estimation of excess mortality, as well as the large number of PLHIV participating in the included studies. However, our study has several limitations, including only searching one database and the screening and inclusion/exclusion criteria being applied by just one author, which may have increased the risk of eligible studies being missed for inclusion. This was due to resource and time constraints to complete the study in time to inform the most recent UNAIDS estimates round. We sought to mitigate these limitations by searching reference of eligible studies for additional studies. Additionally, data extraction was independently checked. Besides age and sex, these studies did not match PLHIV with the general population on socio‐economic status or factors that are important predictors of mortality [47]. The definition of HIV/AIDS‐related mortality and how this was captured varied between studies, and some studies may have underestimated or overestimated HIV/AIDS‐related mortality due to misclassified ICD codes. The reporting for most studies did not allow us to separate out the deaths where all data on cause of death were missing—distinct from deaths where data were available, but the cause was coded as unknown—which may have led to the proportion of deaths due to AIDS being under‐estimated. For studies where data on the exact mortality rates among the general population were unavailable, our method for estimating the mortality rate in the general population by dividing the mortality rate among PLHIV by the SMR will likely have led to some bias as these estimations will differ slightly from the actual values. The percentage of PLHIV on ART was not always available or was unclear, which may also affect the generalizability and interpretation of these findings. While most studies likely had high percentages of PLHIV on ART by the end of their follow‐up (if not the start), only one study had all PLHIV on ART—the study with the lowest overall mortality rates.

### 4.3 Generalizability

No eligible studies were identified from sub‐Saharan Africa or any low‐ or middle‐income countries, so these findings are unlikely to be generalizable to PLHIV on ART outside of Europe, North America and high‐income countries in Asia‐Pacific. Although no data were included from the United States, the results are likely generalizable to PLHIV there, but it is worth considering the differing health insurance policies and health system in the United States from other high‐income countries. When considering other regions, the epidemiology of HIV and the sources of mortality among PLHIV will likely differ. First, there is a high burden of tuberculosis in sub‐Saharan Africa and other regions such as Eastern Europe, unlike in the studies included in this review. Furthermore, all studies included were in settings where sex between men was the major route of transmission, whereas in higher‐burden settings, HIV transmission is mostly through other modes, including injecting drug use and heterosexual intercourse. Finally, the median age of PLHIV in different settings will vary, which will influence the types of mortality experienced.

### 4.4 Implications

That half of the excess mortality among PLHIV in high‐income settings, compared to the general population, is due to non‐AIDS causes shows the need for HIV care to be linked to care for comorbidities, substance use, and, as PLHIV age, geriatric care. Comorbidities are a major source of mortality among PLHIV and often PLHIV have several comorbidities alongside HIV [49]. Our study highlights that a shift in thinking is needed away from excess mortality among PLHIV being entirely due to AIDS, with much of it being due to causes seemingly unrelated to AIDS. There are multiple potential reasons that half of the excess mortality among PLHIV in high‐income countries is non‐AIDS‐related, including PLHIV having higher rates of developing comorbidities like cancers [11] and higher use of substances [46]—these factors are likely interlinked. This shift in thinking is also required for the UNAIDS models reporting all excess mortality among PLHIV as AIDS‐related mortality, as, clearly, this is no longer the case for PLHIV on ART in high‐income countries. The outputs of these models are very influential, as they are used to report the headline figures of the number of AIDS‐related deaths worldwide, by region, and by country, particularly for capturing progress in controlling the worldwide HIV epidemic. The UNAIDS Reference Group on Estimates, Modelling, and Projections has recommended investigating potential modifications to the Spectrum model to estimate AIDS‐related mortality within the envelope of estimated excess mortality among PLHIV [50]. Along with any model changes, UNAIDS will need to consider how to communicate these findings, as this excess non‐AIDS mortality can be reduced and should not be ignored. For mortality among PLHIV who are not on ART, no data were identified on the percentage of excess mortality compared with the general population that was due to AIDS. Therefore, in UNAIDS’ models, continuing to refer to all excess mortality among PLHIV who are not on ART as AIDS‐related is recommended until stronger evidence emerges that indicates otherwise.

While our review found that 53% of excess mortality among PLHIV in high‐income settings is due to AIDS‐related causes, this percentage could be even lower now that more PLHIV are on better regimens and are starting treatment earlier than in our study's median mid‐year of 2008. Overall, there was little data available on cause‐specific excess mortality among PLHIV, and while data are available on cause‐specific mortality in studies from low‐ and middle‐income studies, we identified no studies with data on cause‐specific excess mortality from low‐ or middle‐income countries, highlighting a research gap. Further studies are required on cause‐specific excess mortality, particularly in countries outside of Western Europe, from which most of the included data were identified. We found that the method used to assign causes of death is likely influential when estimating the contribution of mortality due to AIDS, so any future studies should carefully consider how AIDS‐related mortality is being defined.

## 5 CONCLUSIONS

As around half of the excess mortality among PLHIV in high‐income regions was non‐AIDS‐related, an emphasis on preventing and treating comorbidities linked to non‐AIDS mortality among PLHIV is required, including those linked to lifestyle factors and ageing. Models and studies, such as those used by UNAIDS, that seek to quantify the amount of mortality among PLHIV that is due to AIDS‐related causes, should determine the percentage of excess mortality that is non‐AIDS‐related to allow for a better understanding of how mortality among PLHIV could be further reduced.

## COMPETING INTERESTS

The authors have no funding or conflicts of interest to disclose.

## AUTHORS’ CONTRIBUTIONS

AT wrote the first draft, searched for the studies and performed the analyses. JA checked the data extraction. AT and JA performed the quality assessment. LFJ, CVS, JWI‐E and AT conceived the initial concept. ELK and RG provided additional data used for analysis. All authors edited the manuscript and provided critical feedback.

## FUNDING

This project was funded by a grant from UNAIDS. AT is funded by the Wellcome Trust under a Sir Henry Wellcome Postdoctoral Fellowship (222770/Z/21/Z). JWI‐E acknowledges funding from UNAIDS and the MRC Centre for Global Infectious Disease Analysis (reference MR/X020258/1), funded by the UK Medical Research Council (MRC), this UK funded award is carried out in the frame of the Global Health EDCTP3 Joint Undertaking.

## Supporting information




**Table S1**: PRISMA 2020 check list
**Figure S1**: Study inclusion flow diagram
**Table S2**: Quality assessment scores from each marker
**Table S3**: Leave‐one‐out analysis of the pooled percentage of excess and overall mortality due to AIDS

## Data Availability

This is a systematic review, so all data have been published. The tables contain all the data used for analyses.
